# Impact of contrast-enhanced mammography in surgical management of breast cancers for women with dense breasts: a dual-center, multi-disciplinary study in Asia

**DOI:** 10.1007/s00330-022-08906-0

**Published:** 2022-07-05

**Authors:** Yonggeng Goh, Chen-Pin Chou, Ching Wan Chan, Shaik Ahmad Buhari, Mikael Hartman, Siau Wei Tang, Celene Wei Qi Ng, Premilla Pillay, Wynne Chua, Pooja Jagmohan, Eide Sterling, Ying Mei Wong, Loon Ying Tan, Han Yang Ong, Huay-Ben Pan, Herng-Sheng Lee, Bao-Hui Hung, Swee Tian Quek

**Affiliations:** 1 Department of Diagnostic Imaging, National University Hospital, 5 Lower Kent Ridge Rd, Singapore, 119074 Singapore; 2 Department of Diagnostic Imaging, Kaohsiung Veterans General Hospital, Kaohsiung, Taiwan; 3 Department of Breast Surgery, National University Hospital, Singapore, Singapore; 4 Department of Pathology and Laboratory Medicine, Kaohsiung Veterans General Hospital, Kaohsiung, Taiwan

**Keywords:** Breast density, Breast neoplasms, Mammography, Contrast media, Clinical decision-making

## Abstract

**Objective:**

To evaluate the impact of pre-operative contrast-enhanced mammography (CEM) in breast cancer patients with dense breasts.

**Methods:**

We conducted a retrospective review of 232 histologically proven breast cancers in 200 women (mean age: 53.4 years ± 10.2) who underwent pre-surgical CEM imaging across two Asian institutions (Singapore and Taiwan). Majority (95.5%) of patients had dense breast tissue (BI-RADS category C or D). Surgical decision was recorded in a simulated blinded multi-disciplinary team setting on two separate scenarios: (i) pre-CEM setting with standard imaging, and clinical and histopathological results; and (ii) post-CEM setting with new imaging and corresponding histological findings from CEM. Alterations in surgical plan (if any) because of CEM imaging were recorded. Predictors CEM of patients who benefitted from surgical plan alterations were evaluated using logistic regression.

**Results:**

CEM resulted in altered surgical plans in 36 (18%) of 200 patients in this study. CEM discovered clinically significant larger tumor size or extent in 24 (12%) patients and additional tumors in 12 (6%) patients. CEM also detected additional benign/false-positive lesions in 13 (6.5%) of the 200 patients. Significant predictors of patients who benefitted from surgical alterations found on multivariate analysis were pre-CEM surgical decision for upfront breast conservation (OR, 7.7; 95% CI, 1.9-32.1; *p* = 0.005), architectural distortion on mammograms (OR, 7.6; 95% CI, 1.3–42.9; *p* = .022), and tumor size of ≥ 1.5 cm (OR, 1.5; 95% CI, 1.0-2.2; *p* = .034).

**Conclusion:**

CEM is an effective imaging technique for pre-surgical planning for Asian breast cancer patients with dense breasts.

**Key Points:**

*• CEM significantly altered surgical plans in 18% (nearly 1 in 5) of this Asian study cohort with dense breasts.*

*• Significant patient and imaging predictors for surgical plan alteration include (i) patients considered for upfront breast-conserving surgery; (ii) architectural distortion lesions; and (iii) tumor size of ≥ 1.5 cm.*

*• Additional false-positive/benign lesions detected through CEM were uncommon, affecting only 6.5% of the study cohort.*

## Introduction

Accurate pre-operative assessment of breast cancer is critical in determining a patient’s management [1]. Overestimation of tumor size and extent can lead to unnecessary *resection of healthy* tissues while underestimation can lead to re-operations due to inadequate tumor margins. Imaging plays a key role in pre-operative assessment and often relies on non-contrast imaging modalities such as full-field digital mammography (FFDM) and ultrasound (US). However, tumor size assessments on FFDM and US have been shown to correlate poorly with eventual histological size [2] and have been reported to underestimate breast cancer size in up to 35% of patients [3]. Hence, several studies have proposed additional imaging with contrast-enhanced modalities, such as contrast-enhanced magnetic resonance imaging (MRI) in pre-operative breast cancer assessment [4–6]. However, in many healthcare settings and institutions, the high costs and general long waiting times for breast MRI render it non-feasible for use in evaluation of all pre-operative staging cases [7].

Contrast-enhanced mammography (CEM) is an emerging contrast-enhanced breast imaging modality which uses the principle of dual-energy subtraction. It has demonstrated results comparable to MRI in many settings of breast imaging [8–12] and has shown promise in pre-operative staging of breast cancer in several studies [13–17] involving the Caucasian population. As Caucasian women’s breast density is comparably lower [18], these results may not be directly translatable to other population settings (e.g., Asian population) with denser breast which could render accurate pre-operative staging more challenging [19, 20]. Eastern Asian countries also have a younger peak age for breast cancer than Western countries [20]. As far as we know, there are only few small sample–sized single-institutional studies supporting the use of CEM in dense breasts (e.g., Asian women) to date [21, 22]. Hence, the impact that has been documented in other populations may not be directly extrapolated to populations with denser breast tissue.

In this study, the authors seek to validate the impact of pre-operative CEM breast cancer assessment on surgical planning in the Asian population with dense breast parenchyma, with hope to potentially extrapolate these findings to other population settings with dense breast parenchyma too. For patients who had clinically significant surgical plan alterations due to CEM, the authors also seek to identify predictors of the changes in hope of facilitating better utilization of resources and decision-making among breast radiologists and surgeons.

## Methods

### Study population

This retrospective study included two cohorts of adult women (≥ 18 years old) who underwent CEM for pre-biopsy evaluation at the Department of Radiology, Kaohsiung Veterans General Hospital (KSVGH), Taiwan, from February 2012 to November 2019 and the Department of Radiology, National University Hospital (NUH), Singapore, from December 2018 to January 2020. CEM examinations were requested as part of clinical routine or of a previously performed prospective study [23]. Any abnormal CEM findings were provided for clinical care of patients. The cohorts were gathered systemically during the study period for both centers, and a total of 810 CEM studies were collected from these two institutions. Patients who did not receive biopsy (*n* = 246), had biopsy or surgical excision for benign or high-risk breast lesions (*n* = 174), were not surgical candidates (e.g., those with metastatic disease receiving salvage or neoadjuvant chemotherapy) (*n* = 42), or had only unilateral CEM (*n* = 148) were excluded from this analysis. Finally, two hundred eligible women with histologically proven breast cancers who had undergone CEM prior to eventual surgery were included in this study.

The study was approved by both local institutional review boards (IRB number KSVGH22-CT2-18 and NHG DSRB 2016/00508), and the need for informed consent was waived. Of the 200 women who participated in our current study, 69 individuals had previously participated in a study that compared the diagnostic value of CEM and breast MRI for suspicious breast lesions before biopsies [23]. There was no discussion of surgical management with CEM in that study. Our current study has a larger cohort for CEM of breast malignancies, and there is no substantial data redundancy between the two studies.

### Methodology and decision-making (pre- and post-CEM) categories

#### Scenario 1 (pre-CEM)

Anonymized FFDM obtained from routine standard-of-care breast imaging investigations for each patient were reviewed on a workstation in a simulated multi-disciplinary team (MDT) setting comprising a breast radiologist and two breast surgeons. Additional non-contrast images (adjunctive digital breast tomosynthesis (DBT) or key images of measured lesions in breast ultrasound) were also obtained if available. The bilateral whole-breast ultrasound studies were performed by experienced sonographers in both KSVGH and NUH sites, and negative ultrasound studies may necessitate rechecking by radiologists. Targeted ultrasounds for symptomatic women were done by surgeons. All available ultrasound examinations were performed as part of routine clinical assessments for symptomatic lesions or abnormal mammograms.

The treatment decision would be made based on retrospective information obtained from (i) images and radiologists’ reports (i.e., number of lesions, tumor site, size, and extent); (ii) clinical records (i.e., age, previous surgical history, clinical findings, etc.); and (iii) histopathology reports of initial core needle biopsy (i.e., cancer subtype, histological grade, and IHC markers).

At scenario 1, the simulated MDT team is blinded to the CEM findings from scenario 2 and eventual patient outcome.

#### Scenario 2 (post-CEM)

Upon initial confirmation of the pre-CEM treatment plan, the MDT would be provided with additional retrospective information such as (i) CEM images and radiologists’ reports (i.e., number of lesions, tumor site, size and extent on CEM); (ii) results of additional imaging work-ups after CEM (i.e., second-look US, breast MRI, etc.); and (iii) histopathology reports for any newly detected BI-RADS 4–5 lesions on CEM (if any). Pre-surgery MRI was only used for wire localization or biopsy of suspicious additional lesions on CEM with no mammographic or sonographic correlate.

At scenario 2, the MDT would finalize a post-CEM treatment decision after review of these additional findings. As scenario 2 only provided add-on images to scenario 1, no wash-out time between case reviews was indicated.

#### Decision-making categories

**Fig. 1:**
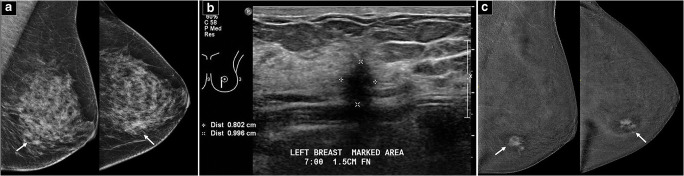
Example of group 1 classification. **a** Full-field digital mammograms (FFDM) of both breasts (CC and MLO views) demonstrate a mass with irregular margins in the left lower inner breast (arrows). **b** Ultrasound of the left breast confirms a suspicious 10-mm hypoechoic mass at the 7OC position, 1.5 cm from the nipple. G2 invasive ductal carcinoma (IDC) (ER/PR+, Her2−) was proven on biopsy. **c** CC and MLO views of contrast-enhanced digital mammogram (CEM) demonstrate identical findings to FFDM and ultrasound. There is a solitary 1.2-cm irregular enhancing mass in the left lower inner breast (arrows). No new findings on CEM were detected (group 1 classification). The patient underwent a wide local excision which revealed a 1.2-cm G2 IDC

**Fig. 2:**
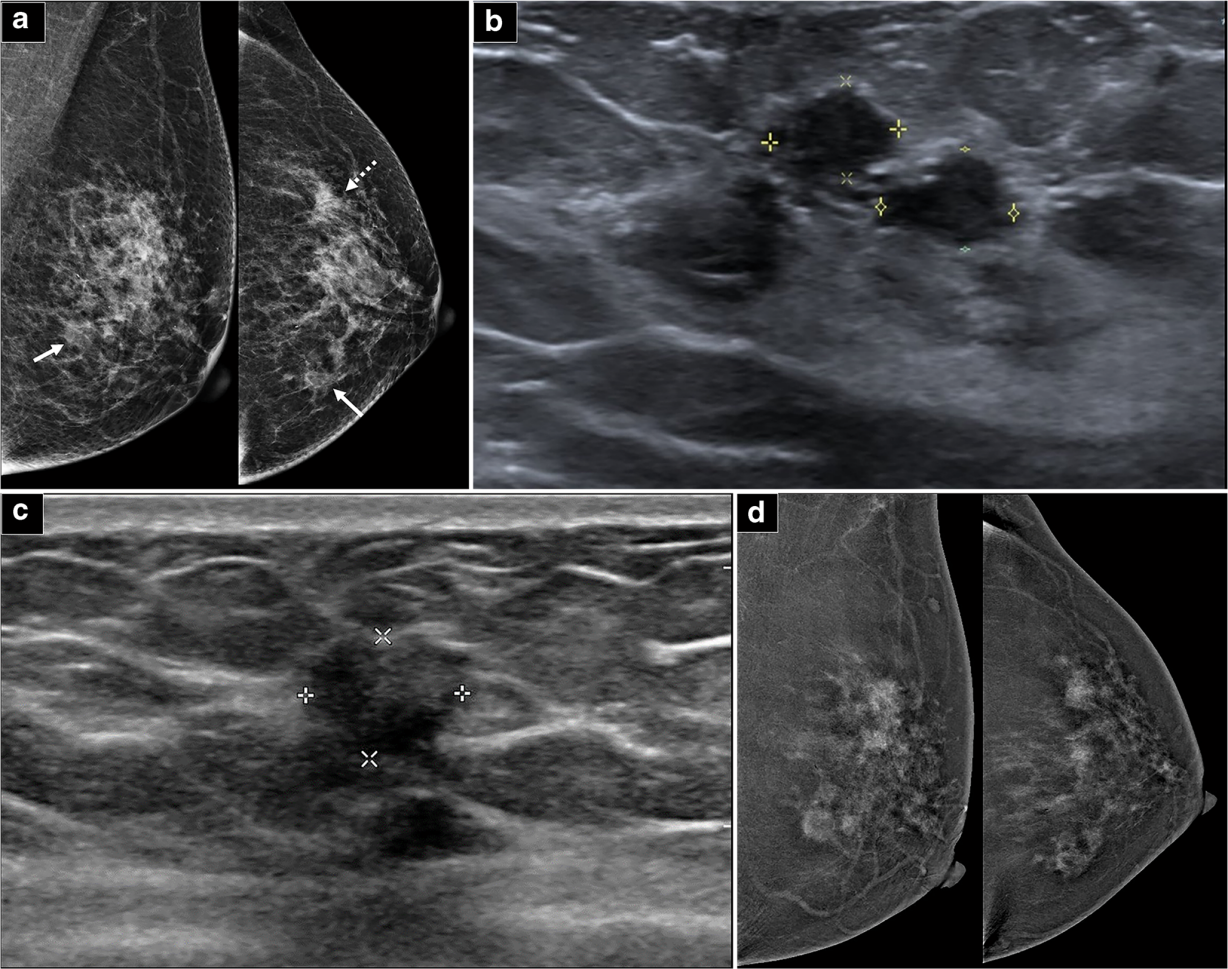
Example of group 2 classification. **a** Full-field digital mammograms (FFDM) of both breasts (CC and MLO views) demonstrate a 10-mm irregular mass in the central inner breast (arrows) and an area of architectural distortion in the left outer breast (dashed arrow). **b** Ultrasound of the left breast confirms 2 small hypoechoic masses in the outer breast, collectively measuring up to 1.2 cm corresponding the area of distortion. **c** Ultrasound of the inner breast also shows a suspicious 10-mm hypoechoic mass at the 9OC position, 4 cm from the nipple. Both masses were biopsied with histology of G1 invasive ductal carcinoma (IDC), triple positive. The patient was scheduled for mastectomy in view of multicentric tumors. **d** CC and MLO views of contrast-enhanced digital mammogram (CEM) performed demonstrate pathological mass and non-mass enhancement of the entire breast. There is significant increase in tumor extent, but this did not alter surgical management as the patient was already scheduled for l mastectomy (i.e., group 2 classification). The right breast (picture not shown) was unremarkable. Mastectomy was eventually performed which revealed IDC with extensive DCIS combined to maximal dimensions of 9.5 cm

**Fig. 3:**
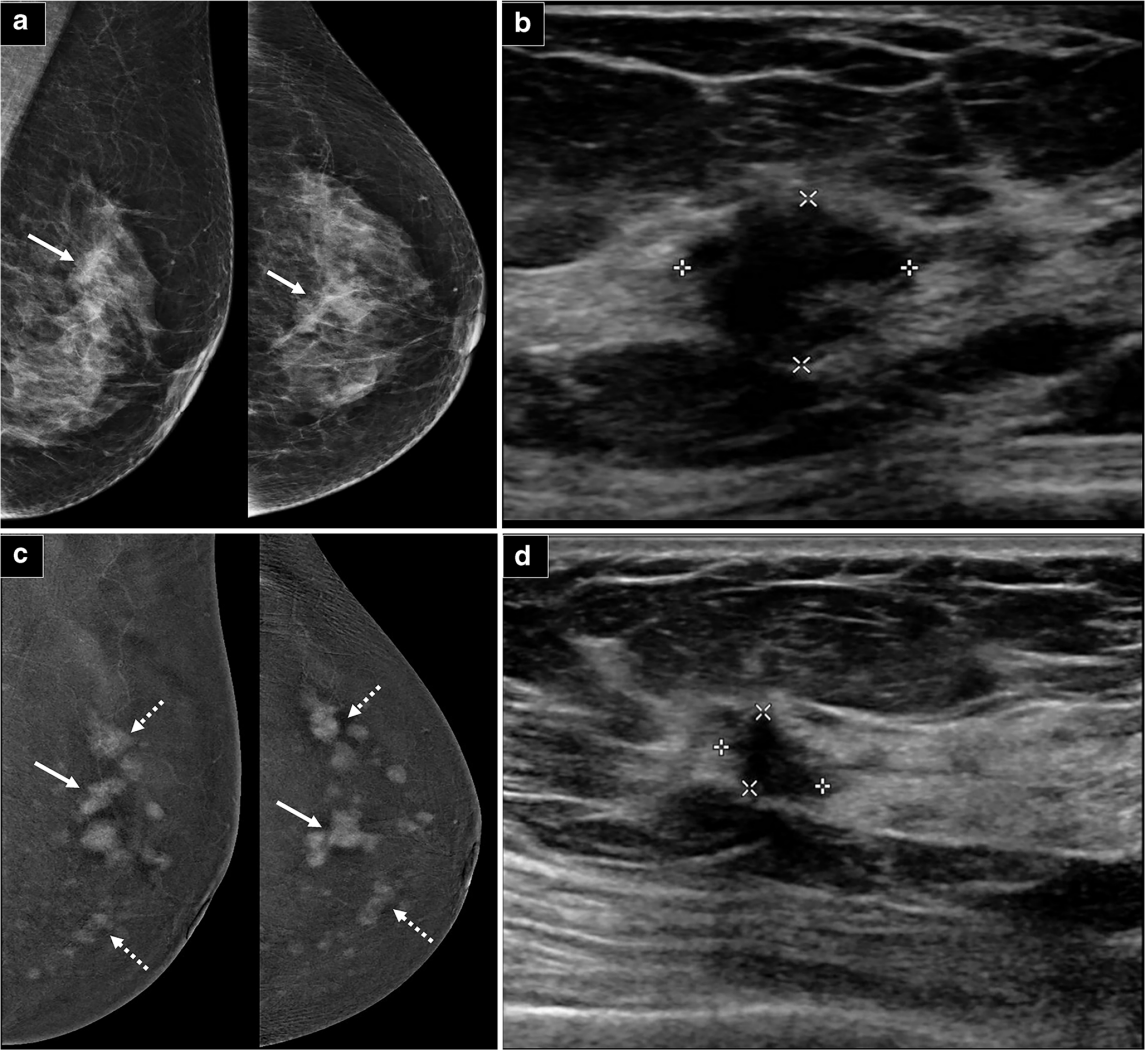
Example of group 3 classification: **a** Full-field digital mammograms (FFDM) of both breasts (CC and MLO views) demonstrate a 14-mm irregular mass in the upper central breast (arrows). **b** Ultrasound of the left breast confirms a 12-mm irregular hypoechoic mass at the 11-12OC periareolar position. **c** CC and MLO views of contrast-enhanced digital mammogram (CEM) performed demonstrate new findings of multiple enhancing tumors of varying sizes in the entire left breast (dotted arrows). The right breast (picture not shown) was unremarkable. **d** A thorough re-look ultrasound revealed several subcentimeter hypoechoic masses (8–9 mm) scattered throughout the breast. A representative 8-mm irregular hypoechoic mass at the 2OC position, 5 cm from the nipple, is demonstrated. The largest/representative lesions were biopsied, and histology results all showed grade 2 invasive lobular carcinoma (ILC), ER/PR+ Her2−. Newly detected cancers that resulted in surgical plan alteration in this case would be classified as group 3

After comparison of pre- and post-CEM treatment plans, the findings were classified into 3 categories:

- Group (1): CEM provided no additional information for surgical management as compared to routine imaging studies (Fig. 1).
- Group (2): CEM provided additional clinical information but did not *alter* the *surgical plan* (e.g., newly detected suspicious lesions/malignancies in a diseased breast undergoing mastectomy) (Fig. 2).
- Group (3): CEM provided additional clinical information which is clinically significant and altered the surgical plan (e.g., newly detected suspicious lesions in a diseased breast undergoing conservation, newly detected suspicious lesions in the contralateral breast or significant change in tumor size (> 2 cm) or extent-to-breast volume ratio from a clinician-perceived perspective) (Fig. 3).

Within group 3, we classified patients with newly detected suspicious lesions which on biopsy were proven benign as false positives. Cases in which significant differences in tumor sizes were observed between CEM imaging and histopathological reports were also evaluated for possible false-positive change (e.g., false upgrade to mastectomy (TM)). Lesions which measured above 1 cm (e.g., 1.5 cm) of the eventual pathologic size were further evaluated in the MDT setting to determine if the overestimation could have resulted in harmful surgical alteration to patients from a clinician-perceived angle. This is due to inherent inter-operator variability in pathologic vs radiologic measurements and limitations of pathologic reference (i.e., gross anatomy of breast tissue changes with fixation and pathologic evaluation) resulting in the inability to achieve perfect imaging-pathological size correlation. However, as a general assumption, lesions which measure ≥ 2 cm of the eventual pathologic size were considered overestimation/false-positive change [24].

All clinical and surgical managements for breast cancer in this study followed the guidelines of the NCCN and the St Gallen 2005 consensus [25, 26]. These decision-making processes were made independently by any of the 2 surgeons present (N.W.Q. (4 years’ experience in breast surgery, T.S.W. (7 years), S.A. (21 years), M.H. (16 years)) in the MDT with any discordant readings resolved by C.C.W. (15 years of experience). Inter-rater agreement between the surgeons in MDT was assessed using Gwet’s kappa.

### CEM image acquisition protocol and image analysis

#### Image acquisition protocol

All CEM images from both institutes were acquired using a mammography system (Selenia Dimensions, Hologic) with dual-energy exposure. The contrast medium Omnipaque 350 (GE Healthcare Inc) was injected into patients via an automatic power injector (Vistron CT injection system, Medrad) at a volume of 1.5 ml/kg of body weight and a rate of 3 ml/s through a peripheral intravenous cannula. After completion, patients were disconnected from the automatic power injector.

The CEM images were obtained starting at 2 min after contrast medium injection. Mediolateral oblique (MLO) and craniocaudal (CC) views of the breast with the lesion of concern would be obtained first, followed by CC and MLO views of the contralateral breast. Two exposures, one at high energy (45–49 kVp) and another at low energy (26–32 kVp), were performed almost simultaneously for each view, and a recombined image between the two was generated to visualize contrast enhancement of both breasts. The image of all 4 views was acquired between 2 and 7 min after contrast agent injection. The pre-surgery bilateral CEM should be performed within 7 min to avoid the influence of greater fibroglandular enhancement on delayed imaging to cancer differentiation in the contralateral breast

#### Image analysis and data parameters

The subtracted CEM images from KSVGH were interpreted by consensus of 3 breast radiologists (C.C.P., H.B.P., and B.H.H.) trained in breast imaging with 17, 27, and 5 years of breast imaging experience, while the CEM images from Singapore were interpreted by consensus of 3 breast radiologists (E.S., G.Y.G., and P.P.) with 6, 5, and 9 years of experience respectively. All readers had more than 3 years of experience in CEM.

In assessing the pre-operative measurements of tumor area/extent, the largest dimension of the mass/area in millimeters was derived from the respective images obtained by each imaging modality (FFDM, US, and CEM). Satellite lesions which were ≤ 0.5 cm apart from the dominant tumor would be grouped together as a single tumor (i.e., bridging tumor). In cases with lesions > 0.5 cm apart (i.e., multifocal (within same quadrant) or multicentric (different quadrants), the lesions would be considered separate lesions instead of a single/grouped lesion.

Potential pre-CEM predictors for patients who benefitted from surgical plan alterations were also collected and reviewed retrospectively. These include:

(i) Patient’s age
(ii) Mammogram density (according to the American College of Radiology Breast Imaging Reporting and Data System (BI-RADS) lexicon)
(iii) Mammographic abnormality (mass/asymmetry, microcalcifications, architectural distortion)
(iv) Size of dominant lesion demonstrated on non-contrast imaging
(v) Number of FFDM–detected lesions
(vi) Tumor histology (invasive (i.e., invasive ductal carcinoma (IDC), invasive lobular carcinoma (ILC), etc.) vs non-invasive tumors (i.e., DCIS)
(vii) Pre-CEM surgical treatment decision (upfront breast conservation vs others (e.g., neoadjuvant chemotherapy (NACT) or mastectomy, etc.))

### Statistical analysis

We performed statistical analyses using Stata version 16. Descriptive statistics for continuous variables were presented as mean ± standard deviation (SD) when normality and homogeneity assumptions were satisfied, whereas for categorical variables, median (IQR) and *n* (%) were reported instead. Inter-rater agreement between the surgeons in MDT was assessed using Gwet’s kappa to account for the paradox effect of a high percentage of no change in decision [27]. Predictors for patients who benefited from surgical plan alterations were evaluated using univariate and multivariate logistic regression using an all variable model. Receiver operating characteristic (ROC) curve analysis was performed for lesion size on FFDM to determine the optimal cutoff value (best balance between sensitivity and specificity).

## Results

### Patient characteristics

Two hundred eligible women (mean age, 54.3 ± 10.2 years) were found across two institutes (119 from KSVGH and 91 from NUH). There was a total of 232 histologically proven breast cancers in these women. Patients’ characteristics and histopathology of breast cancers are shown in Table 1. Imaging studies performed at the time of clinical routine for these patients are as described: DBT and bilateral whole-breast ultrasounds were present in 51.5% of the study population (71 patients from KSVGH and 32 patients from NUH), while the remaining patients had a mixture of non-contrast studies performed (e.g., targeted US instead of whole-breast US) due to intra-individual preferences among breast surgeons and radiologists.

**Table 1:** Baseline characteristics of patients (*n* = 200)

Parameter	Value (percentage)
Participant characteristics	
Age, years	
Mean ± SD	53.4 ± 10.2
Range	29–81
Breast density (based on FFDM and ACR-BI-RADS lexicon)	
A (entirely fatty)	1/200 (0.5%)
B (scattered fibroglandular)	8/200 (4.0%)
C (heterogeneously dense)	160/200 (80.0%)
D (extremely dense)	31/200 (15.5%)
Patient’s history of breast cancer	
Family history	33/200 (16.5%)
Personal history	10/200 (5.0%)
Tumor distribution	
No. of patients with unifocal cancer	172/200 (86.0%)
No. of patients with 2 tumors	24/200 (12.0%)
No. of patients with ≥ 3 tumors^#^	4/200 (2.0%)
Total no. of tumors identified in 200 subjects	232^##^
Histology*	
IDC (Invasive ductal carcinoma)	108/200 (54.0%)
Grade 1	22/108 (20.4%)
Grade 2	54/108 (50.0%)
Grade 3	32/108 (29.6%)
DCIS (ductal carcinoma in situ)	60/200 (30.0%)
Low grade	10/60 (16.7%)
Intermediate grade	25/60 (41.7%)
High grade	25/60 (41.7%)
ILC (invasive lobular carcinoma)	24/200 (12.0%)
Others (i.e., mucinous cancer, papillary cancer, tubular carcinoma, etc.) or mixed cancers (e.g., mixed IDC with ILC)	8/200 (4.0%)
Tumor markers for invasive tumors	
Luminal A	92/140 (65.7%)
Luminal B	21/140 (15.0%)
Triple negative	11/140 (7.9%)
Her2 enriched	16/140 (11.4%)

^#^Patients with > 3 tumors identified are counted as a maximum of 3 in this study

*Only histology of the dominant tumor in patients with > 1 tumor is expressed

^##^ER and PR were considered positive if larger than 10% of the stained nuclei. Cut-off point for Ki67 was 20%. HER2 expression was considered positive if amplified with fluorescent in situ hybridization

### Surgical management change

CEM did not provide any additional information in 143 patients (group 1); it revealed additional information in 8 patients, but the information did not *alter surgical plans* (group 2). Among 49 patients in group 3, thirteen patients (26.5%) had newly detected CEM lesions which led to 15 additional biopsies with benign results. Of all 200 patients, 164 (82.0%) did not have their surgical management altered post-CEM. For the remaining 36 (18.0%) patients who have had altered surgical plans, 24 had clinically significant larger tumor size/extent (> 2 cm) on CEM, and 12 had additional cancer (total of 18) detected by CEM. Among the 12 patients with additional cancer detected, 4 had ≥ 2 lesions detected and 3 had contralateral synchronous breast cancers. The histologies for the additional detected benign and malignant lesions after CEM exams are summarized in Table 2. Of the 18 additional malignant lesions on CEM, 11 revealed an IDC, 6 an ILC, and 1 a ductal carcinoma in situ. In contrast, CEM had shown less additional lesions which were benign on histology (i.e., 15 benign vs 18 malignant).

**Table 2:** Histology results for newly detected lesions by CEM (*n* = 33)

Parameter	Value
Benign (*n* = 15 from 13 patients)	
Histology	
Fibrocystic change, sclerosing adenosis, stromal fibrosis	8/15 (53.3%)
Fibroadenoma	5/15 (33.3%)
Papilloma	1/15 (6.7%)
Radial scar	1/15 (6.7%)
Biopsy modality for benign lesions	
Ultrasound guided	11/15 (73.3%)
MRI guided	4/15 (26.7%)
Malignant (*n* = 18 for 12 patients)	
Histology	
Invasive ductal carcinoma	11/18 (61.1%)
Invasive lobular carcinoma	6/18 (33.3%)
Ductal carcinoma in situ	1/18 (5.6%)
Biopsy modality for malignant lesions	
Ultrasound guided	17/18 (94.4%)
MRI guided	1/18 (5.6%)

Of all patients with altered surgical plans, surgical-pathological correlation could not be determined for 5 of them due to insufficient data (2 refused surgery, 1 sought treatment in a different institution, and 2 had conversion to NACT). All remaining 31 patients, for whom final surgical-pathological correlations were determined, had meaningful surgical change (wider excision or conversion to mastectomy) as deemed by the MDT. Twenty-eight (90.3%) of them had lesion size within 1 cm of pathological size; there was no false surgical upgrade (i.e., no case with lesion size ≥ 2 cm of eventual pathologic size). The changes in surgical managements for these 31 patients are summarized in Table 3.

**Table 3:** Change in surgical management based on CEM in 31 patients

Treatment change	No. (percentage)
Wider excision (2 cm larger than initial plan)	5/31 (16.1%)
Wide excision to mastectomy	23/31 (74.2%)
Contralateral surgery	3/31 (9.7%)

**Fig. 4:**
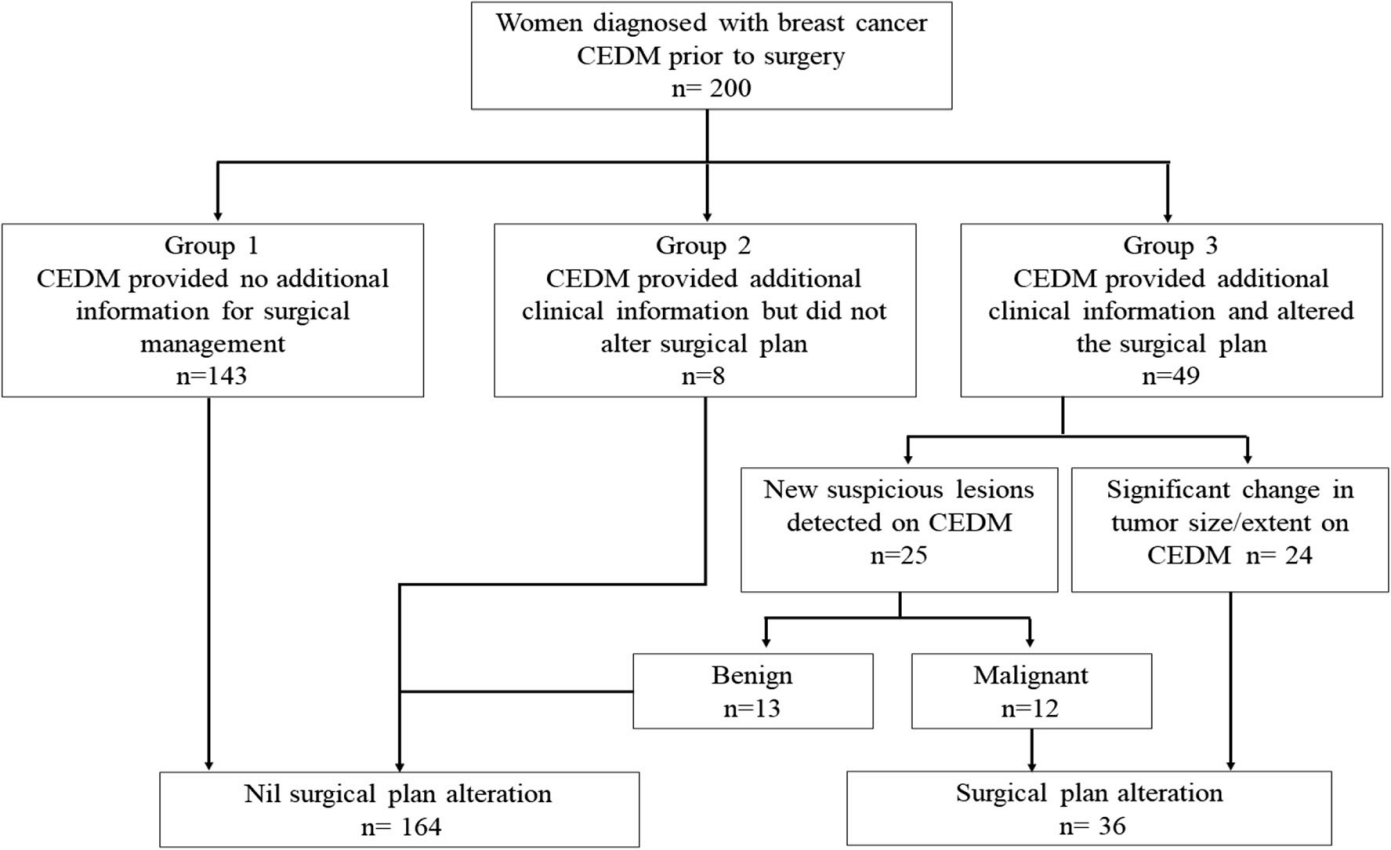
Flowchart of CEDM Cohort Categories and Respective Results

The overall results are summarized in a flowchart (Fig. 4). The overall agreement between surgeons for surgical management plans and determination of possible false-positive/tumor upgrades were 0.906 and 1.0, respectively.

### Predictors for benefit from CEM

Overall, CEM *resulted in* significantly *altered surgical plans* for 18% (36/200) of the study cohort. At univariate analysis, no predictors were found significantly associated with change in surgical management, apart from pre-CEM decision. However, multivariate analysis showed that changes in surgical management after CEM were significantly associated with the (1) pre-CEM decision for upfront breast-conserving surgery (i.e., wide excision with/without flap reconstruction) (OR, 7.7; 95% CI, 1.9-32.1; *p* = 0.005); (2) mammographic finding of architectural distortion (OR, 7.6; 95% CI, 1.3–42.9; *p* = 0.022); and (3) tumor size of ≥ 1.5 cm on FFDM (OR, 1.5; 95% CI, 1.0-2.2; *p* = 0.034). The optimal cutoff size of 1.5 cm was obtained from a ROC curve analysis (sensitivity, 88.6%; specificity, 67.8 %). The above findings are summarized in Table 4.

**Table 4:** Association between surgical management change and pre-CEM predictors on CEM

			Unadjusted analysis		Adjusted analysis	
Parameter	No change in surgical management	Change in surgical management	Odds ratio (95% CI)	*p* value	Odds ratio (95% CI)	*p* value
Age (± SD)	53.3 ± 10.1	53.6 ± 10.9	1.00 (0.97–1.04)	0.878	1.03 (0.98–1.08)	0.240
Mammogram density						
Fatty + scattered fibroglandular tissue	8 (88.9%)	1 (11.1%)	1.0	–	1.0	–
Heterogeneously dense + extremely dense	156 (81.7%)	35 (18.3%)	1.8 (0.2–14.8)	0.587	2.4 (0.2–24.4)	0.459
Mammogram abnormality						
Mass/asymmetry	83 (84.7%)	15 (15.3%)	0.90 (0.39–2.11)	0.815	1.9 (0.4–9.4)	0.413
Microcalcifications	55 (83.3%)	11 (16.7%)	1.0	–	1.0	–
Architectural distortion	17 (65.4%)	9 (34.6%)	2.6 (0.9–7.5)	0.065	7.6 (1.3–42.9)	0.022*
Occult	9 (90%)	1 (10%)	0.56 (0.06–4.84)	0.595	NA	NA
No. of mammogram-detected lesions						
0 or 1 (i.e., unifocal)	141 (81.5%)	32 (18.5%)	1.0	–	1.0	–
> 1 (multifocal/multicentric)	23 (85.2%)	4 (14.8%)	0.77 (0.25–2.37)	0.644	1.7 (0.4–7.7)	0.504
Size of dominant abnormality on FFDM						
≥ 1.5 cm	97 (80.2%)	24 (19.8%)	1.4 (0.6–3.0)	0.404	4.6 (1.3–15.7)	0.015*
< 1.5 cm	67 (84.8%)	12 (15.2%)	1.0	–	1.0	**–**
Histology						
Invasive lobular carcinoma (ILC)	19 (79.2%)	5 (20.8%)	1.17 (0.36–3.82)	0.79	0.60 (0.36–9.94)	0.722
Other solid tumors (i.e., IDC, etc.)	96 (82.8%)	20 (17.2%)	0.93 (0.41–2.09)	0.86	0.73 (0.58–9.36)	0.812
Non-solid tumors (i.e., DCIS)	49 (81.7%)	11 (18.3%)	1.0	–	1.0	–
Hormone profile of detected cancers						
Luminal A	74 (78.7%)	20 (21.3%)	2.6 (0.6–12.0)	0.230	2.7 (0.5–13.5)	0.240
Luminal B	19 (90.5%)	2 (9.5%)	1.0	–	1.0	–
Triple negative	10 (90.9%)	1 (9.1%)	0.95 (0.08–11.8)	0.968	1.1 (0.1–21.1)	0.931
Her2 enriched	14 (87.5%)	2 (12.5%)	1.4 (0.2–10.8)	0.773	2.0 (0.2–19.4)	0.540
Pre-CEM decision						
Breast conservation (wide local excision with/without flap reconstruction)	91 (75.8%)	29 (24.2%)	3.3 (1.4–8.0)	0.008**	7.7 (1.9–32.1)	0.005**
Others (e.g., neoadjuvant chemotherapy (NACT) + total mastectomy (TM) or bilateral surgery)	73 (91.3%)	7 (8.8%)	1.0	–	1.0	–

Significant *p* value in bold (*p < 0.05, **p < 0.01, and ***p < 0.001)

*NA* not available due to 0 count

### Comparison of CEM vs MRI

MRI was performed for 96/200 (48.0%) patients in this study. All suspicious lesions detected on CEM had corresponding findings on MRI, and there were no CEM occult lesions which were subsequently detected on MRI. MRI–guided procedures were performed on 5/96 (5.2%) patients for lesions detected on CEM which had no sonographic or mammographic correlate. This yielded 1 malignant (i.e., DCIS) and 4 benign results as summarized in Table 2.

## Discussion

In this study, CEM resulted in significantly altered surgical plans in 18% (36/200) of the study cohort. This result is identical to that of several other previous CEM studies published to date. Ahsberg et al [13, 14], Ali-Mucheru et al [13, 14], and Bicchierai et al [16] found significantly altered surgical plans in 21% (10/47), 20% (20/101), and 18.4% (60/326) of their patients, respectively. This finding suggests that CEM can be a valuable tool in the pre-operative assessment of breast cancers in the Asian population despite the known challenges of staging in Asian women who have denser breast tissue than the Western population [19]. These findings could also be potentially translatable to other population settings with dense breast tissue as a very high proportion of our study cohort (191/200, 95.5%) have dense breasts. In addition, CEM caused no significant patient harm as there was no false surgical upgrade or clinically significant under/overestimation of tumor size and/or extent in our study cohort. As benign lesions can enhance on CEM, it is inevitable to have false-positive lesions resulting in additional benign biopsies. The unnecessary biopsies could have been amplified in our study cohort as half of our patients are under the age of 50. After menopause, the relative age-specific incidence of benign breast diseases tends to decrease. Despite these factors, the false-positive rate was noted to be low at 6.5% in our study cohort. However, despite the low risk, the possibility of requiring additional benign biopsies and imaging examinations should still be conveyed to patients once CEM becomes more mainstream as a pre-surgical planning imaging modality.

As CEM did not provide results in surgical plan alterations in 82.0% (164/200) of the study cohort, the authors sought to identify predictors in patients who benefited from surgical plan alterations. This could help to facilitate better utilization of resources and decision-making for breast radiologists and surgeons in the future. These predictors were assessed using multivariate analysis which revealed 3 predictors with statistical significance (*p* < .05): (1) size of lesion ≥ 1.5 cm on FFDM; (2) mammographic finding of architectural distortion (AD); and (3) pre-CEM surgical decision for upfront breast conservation.

Size of lesion (≥ 1.5 cm) has a significant association with beneficial surgical plan alteration after CEM imaging, with an OR of 4.6 (95% CI, 1.3, 15.7; *p* = .015). This finding is in line with clinically/epidemiologically observed findings as T1 breast tumors (≤ 2.0 cm) have been shown to carry good prognosis with cancer-specific survival rates of 90–95% after 5–10 years [28]. These small lesions are commonly detected in asymptomatic patients and are unlikely to be extensive [29]. Hence, size estimations of small lesions detected on routine breast imaging studies are expected to be accurate and additional imaging with CEM may not be necessary in such cases. CEM could therefore be reserved for imaging lesions which are slightly larger, the minimum size for which should be approximately 1.5 cm, as shown by our ROC curve analysis.

Secondly, the mammographic finding of AD lesions also showed a significant association with beneficial surgical decision change in our study, with an OR of 7.6 (95% CI, 1.3–42.9; *p* = .022). This is likely due to the difficulty/subjectivity in estimating the true size and extent of AD on FFDM, for which CEM has already been shown to provide accurate characterization and size estimation [30]. As size/extent estimation of AD is more challenging as compared to that of other masses or microcalcifications, CEM could play a major role in pre-surgical planning for breast cancer patients with a primary finding of AD.

Thirdly, ILC accounted for 33% of all additional breast cancers detected on pre-surgery CEM in this study. ILC is associated with a higher rate of multifocal, multicentric, and contralateral disease than IDC. Pre-surgical MRI for ILC has shown benefit in patients with ILC by altering surgical plans in 25–39% of patients [15, 31] as MRI has been shown to be superior to non-contrast modalities (e.g., FFDM and ultrasound) in ILC detection and size measurement [32]. CEM, a potential MRI surrogate, has demonstrated its utility in detecting occult ILC and altering the surgical plans in this Asian women study.

Lastly, pre-CEM surgical decision for upfront breast conservation showed the strongest association with beneficial surgical plan alteration in our study, with an OR of 7.7 (95% CI, 1.9-32.1; *p* = 0.005). Compared to Western countries, Asia has traditionally reported high mastectomy rates and low breast-conserving surgery rates among early breast cancer patients [33]. This is not an unexpected finding as patients who undergo upfront breast-conserving surgery have more potential possibilities of a surgical change as compared to patients with mastectomy or NACT. However, while CEM may not be as useful in patients who are not undergoing upfront breast conservation, the accuracy of CEM staging and high negative predictive value [34] can provide surgeons and radiologists with more surgical and diagnostic confidence, respectively.

Our study has limitations. Firstly, there is no dedicated/standardized recruitment criteria in this retrospective study. For example, the selection of patients for CEM were initiated by either the surgeons or radiologists in this study with reasons that could be influenced by clinicians’ preference, experience, and intuition. These factors would inevitably result in patient diversity. Also, there is inter-operator variability in types and numbers of imaging modalities used in pre-surgical planning among radiologists (e.g., FFDM/DBT +/− US (whole breast vs targeted only) in the two study institutions. The heterogeneity of investigations used could impair the strength of the study. Nonetheless, the results obtained are fairly similar to those of other published studies [13, 14] and reflect the common practice of pre-surgical evaluation in these Asian countries. Secondly, there is also a lack of long-term follow-up data for a small number of patients (e.g., patients recruited from 2019 to 2020) in this cohort. Hence, for these patients, benign-looking findings on CEM were follow-up only 1 year within the study time frame.

## Conclusion

CEM is an effective imaging tool in pre-surgical planning for women with dense breast parenchyma. Pre-CEM surgical decision for upfront breast conservation, architectural distortion lesions, and tumor size of ≥ 1.5 cm on FFDM have the highest association with beneficial surgical plan change after CEM.

## Abbreviations

AD: Architecture distortion
BCS: Breast-conserving surgery
CEM: Contrast-enhanced mammography
DBT: Digital breast tomosynthesis
DCIS: Ductal carcinoma in situ
FFDM: Full-field digital mammography
IDC: Invasive ductal carcinoma
ILC: Invasive lobular carcinoma
MRI: Magnetic resonance imaging
TM: Total mastectomy
US: Ultrasound

## Declarations

### Guarantor

The scientific guarantor of this publication is Dr. Goh Yonggeng.

### Statistics and biometry

Ms. Yap Qai Wen from the National University of Singapore (NUS) Medicine Biostatistics Unit for assistance in biostatistics kindly provided statistical advice for this manuscript.

### Study subjects or cohorts overlap

There are subject/cohort overlap for the patients enrolled in this current study with a previous study that compared CEM and breast MRI (Eur J Radiol. 2015 Dec;84(12):2501-8).

Of the 200 women who participated in our current study, 69 individuals had previously participated in a study that compared the diagnostic value of CEM and breast MRI for suspicious breast lesions before biopsies. There was no discussion of surgical management with CEM in the previous study. Our current study has a larger cohort for CEM of breast malignancies, and there is no substantial data redundancy between the two studies.

The IRB full names and approval numbers are as follows:

This study at KSVGH: KAOHSIUNG V.G.H. Institutional Review Board. IRB No: KSVGH22-CT2-18.

Early study at KSVGH in 2012: KAOHSIUNG V.G.H. Institutional Review Board. IRB No: VGHKS11-CT4-16.

NUH: “National Healthcare Group Domain Specific Review Board” (NHG DSRB).

Approval number: 2016/00508.

### Methodology

• retrospective

• cross-sectional study

• multicenter study
